# Functional Identification of Novel Cell Death-inducing Effector Proteins from *Magnaporthe oryzae*

**DOI:** 10.1186/s12284-019-0312-z

**Published:** 2019-08-06

**Authors:** Xinrui Guo, Debin Zhong, Wei Xie, Yanhua He, Yueqin Zheng, Yan Lin, Zaijie Chen, Yijuan Han, Dagang Tian, Wende Liu, Feng Wang, Zonghua Wang, Songbiao Chen

**Affiliations:** 1 Institute of Oceanography, Marine Biotechnology Center, Minjiang University, Fuzhou, 350108 China; 2 Fujian Academy of Agricultural Sciences, Biotechnology Research Institute, Fuzhou, 350003 China; 3 State Key Laboratory of Ecological Pest Control for Fujian and Taiwan Crops, College of Plant Protection, Fujian Agriculture and Forestry University, Fuzhou, China; 4 State Key Laboratory for Biology of Plant Diseases and Insect Pests, Institute of Plant Protection, Chinese Academy of Agricultural Sciences, Beijing, 100193 China

**Keywords:** *Magnaporthe oryzae*, Rice, Effector, Cell death, Immune response

## Abstract

**Background:**

Secreted effector proteins play critical roles in plant-fungal interactions. The *Magnaporthe oryzae* genome encodes a large number of secreted proteins. However, the function of majority of *M. oryzae* secreted proteins remain to be characterized. We previously identified 851 in planta-expressed *M. oryzae* genes encoding putative secreted proteins, and characterized five *M. oryzae* cell death–inducing proteins MoCDIP1 to MoCDIP5. In the present study, we expand our work on identification of novel MoCDIP proteins.

**Results:**

We performed transient expression assay of 98 more in planta-expressed *M. oryzae* putative secreted protein genes, and identified eight novel proteins, MoCDIP6 to MoCDIP13, that induced plant cell death. Yeast secretion assay and truncation expression analysis revealed that the signal peptides that led the secretion of proteins to the extracellular space, were required for cell death inducing activity of the novel MoCDIPs except for MoCDIP8. Exogenous treatment of rice seedlings with recombinant MoCDIP6 or MoCDIP7 resulted in enhanced resistance to blast fungus, indicating that the two MoCDIPs trigger cell death and elicit defense responses in rice.

**Conclusions:**

The newly identified MoCDIP6 to MoCDIP13, together with previously identified MoCDIP1 to MoCDIP5, provide valuable targets for further dissection of the molecular mechanisms underlying the rice-blast fungus interaction.

**Electronic supplementary material:**

The online version of this article (10.1186/s12284-019-0312-z) contains supplementary material, which is available to authorized users.

## Background

Plant pathogenic fungi are the causal agents of many of the world’s most destructive plant diseases, causing serious agricultural losses worldwide. Plant pathogenic fungi have diverse lifestyles and interact with host plants in various ways: biotrophic fungi colonize and obtain nutrients from living host tissue; necrotrophic fungi infect host tissue and harvest nutrients from dead host cells; whereas hemibiotrophic fungi combine an initial biotrophic phase with a subsequent necrotrophic phage (Lo Presti et al. 2015). Despite the diversity of interaction manners, all plant pathogenic fungi secrete extracellular proteins to facilitate infection. These secreted proteins may function in the apoplast as virulence factors, toxins, and degradative enzymes, or within the plant cytoplasm to manipulate host cell physiology and suppress host immune response (Giraldo and Valent 2013; Kim et al. 2016). In turn, plants have evolved sophisticated immune systems to protect themselves from pathogen invasion, including a basal defense system that recognizes conserved pathogen-associated molecular patterns (PAMPs), and a second layer of immunity through the recognition of secreted effector proteins for triggering defense responses (Jones and Dangl 2006).

The filamentous ascomycete *Magnaporthe oryzae* causes the most devastating blast disease on rice and other cereal crops including wheat (Ebbole 2007; Dean et al. 2012). *M. oryzae* is a hemibiotrophic fungal pathogen that invades host cells biotrophically and grows necrotrophically on dead tissues. Based on genome sequences, *M. oryzae* encodes a large number of putative secreted proteins (predicted between 739 to 2,470) (Dean et al. 2005; Yoshida et al. 2009; Choi et al. 2010). More than 43 secreted proteins have been functionally identified, including 10 avirulence (Avr) proteins, PWL1, PWL2 (Kang et al. 1995; Sweigard et al. 1995), AvrPi-ta (Orbach et al. 2000), AvrPiz-t (Li et al. 2009), Avr-Pia, Avr-Pii, Avr-Pik/km/kp (Yoshida et al. 2009), Avr-CO39 (Cesari et al. 2013), AvrPi9 (Wu et al. 2015), and AvrPib (Zhang et al. 2015; Zhang et al. 2018); four biotrophy-associated secreted proteins, BAS1 to BAS4 (Mosquera et al. 2009); five secreted proteins that are required for pathogenicity, MPG1 (Talbot et al. 1993), EMP1 (Ahn et al. 2004), MHP1 (Kim et al. 2005), Slp1 (Mentlak et al. 2012), and MC69 (Saitoh et al. 2012); 12 suppressors of plant cell death proteins, IUG6, IUG9, NUP1, NUP2 and NUP3 (Dong et al. 2015), MoHEG13 (Mogga et al. 2016), and SPD2, SPD4, SPD7, SPD8, SPD9 and SPD10 (Sharpee et al. 2017); and 12 plant cell death-inducing proteins, MoHrip1 (Chen et al. 2012), MoCDIP1 to MoCDIP5 (*M. oryzae* cell death-inducing proteins) (Chen et al. 2013), MoHrip2 (Chen et al. 2014), MSP1 (Wang et al. 2016), MoNLP1, MoNLP2 and MoNLP4 (Fang et al. 2017), and MoSM1 (Hong et al. 2017). Over the past decades, advances in functional identification of secreted effector proteins from *M. oryzae* have remarkably enhanced our understanding of the molecular mechanisms involved in rice-*M. oryzae* interactions (Liu et al. 2014; Tang et al. 2017).

In a previous study, we performed transcriptome analyses on blast-infected rice leaves, and identified 851 in planta-expressed *M. oryzae* genes encoding putative secreted proteins. We performed transient expression of 42 in planta-expressed putative secreted protein genes, and identified five *M. oryzae* cell death–inducing proteins (MoCDIP1 to MoCDIP5), that induced cell death in plant cells (Chen et al. 2013). In the present study, we expand our work on identification of novel MoCDIP proteins. We cloned 98 more in planta-expressed *M. oryzae* putative secreted protein genes, and identified eight novel putative proteins, MoCDIP6 to MoCDIP13, that induced cell death in plant cells. We further demonstrated that exogenous treatment of rice seedlings with recombinant MoCDIP6 and MoCDIP7 resulted in enhanced resistance to rice blast, indicating that the two MoCDIP proteins trigger cell death and elicit defense responses in rice.

## Results

### Identification of eight *M. oryzae* cell death-inducing proteins

**Fig. 1:**
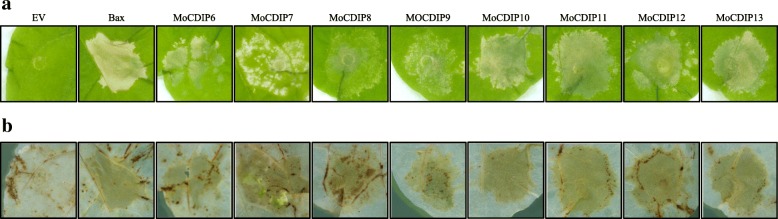
Identification of eight novel *M. oryzae* cell death-inducing proteins (MoCDIPs). **a** Transient expression assay of 98 *M. oryzae* putative secreted proteins revealed that eight of them induced cell death in *N. benthamiana* leaves. EV, an empty vector pGD. **b** Transient expression of the eight *M. oryze* cell death-inducing proteins induced H_2_O_2_ accumulations in *N. benthamiana* leaves as shown by 3,3′-Diaminobenzidine tetrachloride (DAB) staining. The leaves were photographed 5 days after infiltration. The agroinfiltration experiments were repeated at least three times

We previously identified five in planta-expressed *M. oryza* secreted proteins, MoCDIP1 to MoCDIP5, as cell death-inducing proteins (Chen et al. 2013). In this study, to get more insight into the function of in planta-expressed *M. oryzae* secreted proteins, we conducted functional characterization of more genes from the previously identified 851-gene list. A total of 98 *M. oryzae* putative secreted protein genes were amplified by PCR (Additional file 1: Table S1) and were cloned into the plant expression vector pGD (Goodin et al. 2002). The *M. oryzae* putative secreted protein genes were investigated through agro-infiltration in leaves of *Nicotiana benthamiana*. Among the 98 gene constructs, eight were found to induce cell death in *N. benthamiana* leaves (Fig. 1a). 3,3′-Diaminobenzidine tetrachloride (DAB) staining further showed the accumulation of H_2_O_2_ in agro-infiltrated *N. benthamiana* leaves which was correlated with cell death lesions (Fig. 1b). The severity of cell death in *N. benthamiana* leaves induced by the eight constructs varied. While MGG_13283 and MGG_14371 induced clear necrosis symptoms at 3 to 4 days after infiltration, the cell death phenotypes induced by MGG_01532, MGG_03354, MGG_05038, MGG_08411, MGG_12275, and MGG_12521, were visible at 4 to 6 days after infiltration, with relatively weak symptoms. The eight putative proteins, MGG_01532, MGG_03354, MGG_05038, MGG_08411, MGG_12275, MGG_12521, MGG_13283, and MGG_14371 were thus referred to as MoCDIP6 to MoCDIP13, respectively (Additional file 1: Table S1).

### *MoCDIP6* to *MoCDIP13* genes are expressed during infection

**Fig. 2:**
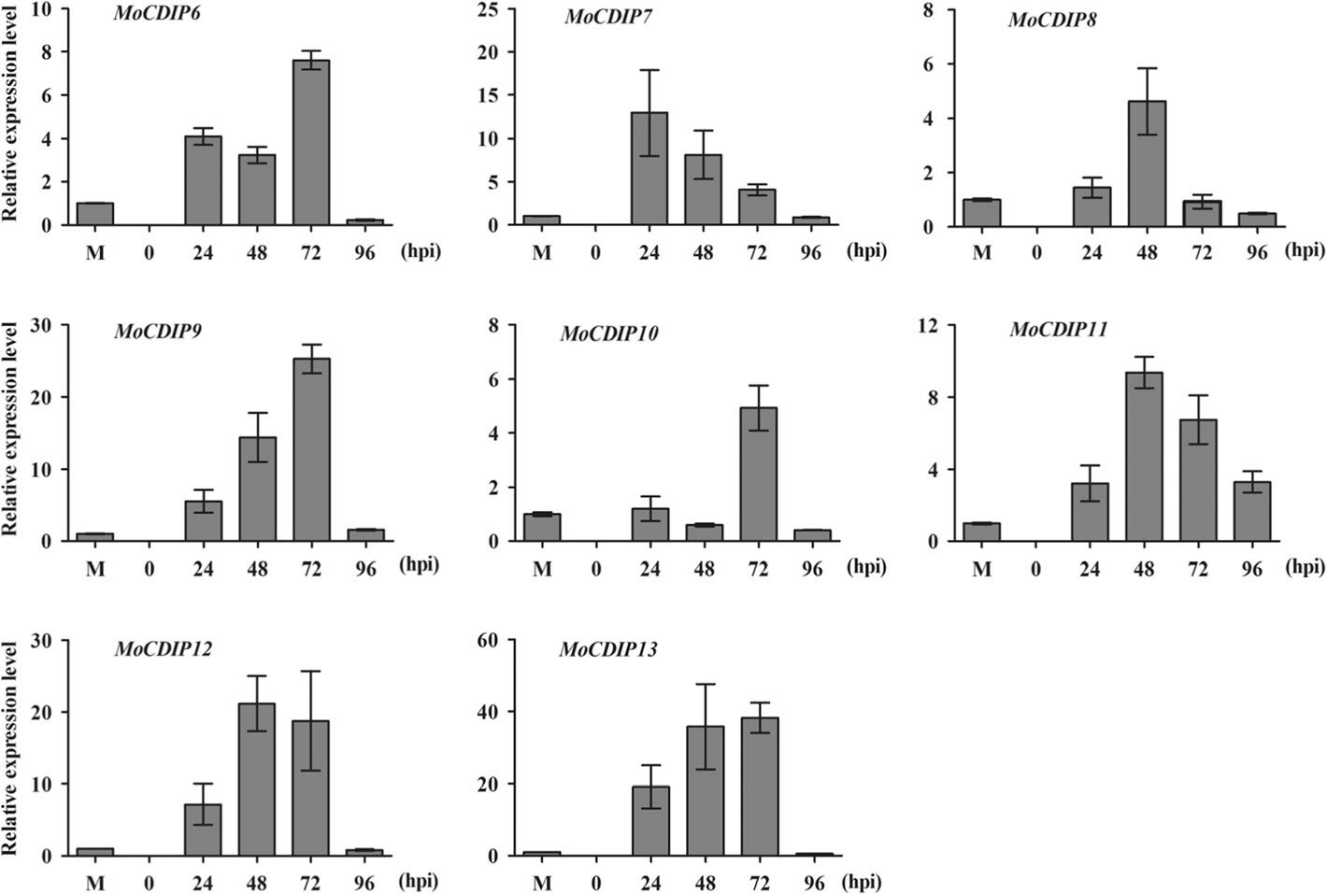
qRT-PCR confirmation of in planta expression pattern of the eight *MoCDIP* genes. Total RNA samples extracted from *M. oryzae* mycelium (M), and from blast-infected rice leaves at 0, 24, 48, 72, or 96 hpi were subjected to qRT-PCR

The eight *MoCDIP* genes, *MoCDIP6* to *MoCDIP13*, were screened from transcriptome profiles of *M. oryzae*-infected rice leaves (Chen et al. 2013). To further confirm the in planta expression pattern of the eight *MoCDIP* genes, a rice cultivar MH3301 was artificially inoculated with a virulent *M. oryzae* isolate 501–3, and quantitative real-time reverse transcription polymerase chain reaction (qRT-PCR) was performed to detect the expression of the eight *MoCDIP* genes in inoculated rice leaves. The results showed that *MoCDIP6* to *MoCDIP13* were expressed at low levels in mycelia. In contrast, the eight *MoCDIP* genes were all expressed at significantly higher levels in *M. oryzae*-inoculated rice leaves (Fig. 2). While relatively higher level of the *MoCDIP7* transcript was detected in inoculated rice leaves at 24 h post-inoculation (hpi), higher expression of the rest *MoCDIPs* were detected in inoculated rice leaves at 48 and/or 72 hpi (Fig. 2). Overall, qRT-PCR results confirmed that the eight *MoCDIP* genes were expressed during infection stages.

### Signal peptides of seven MoCDIPs are required for cell death-inducing activity in *N. benthamiana*

**Fig. 3:**
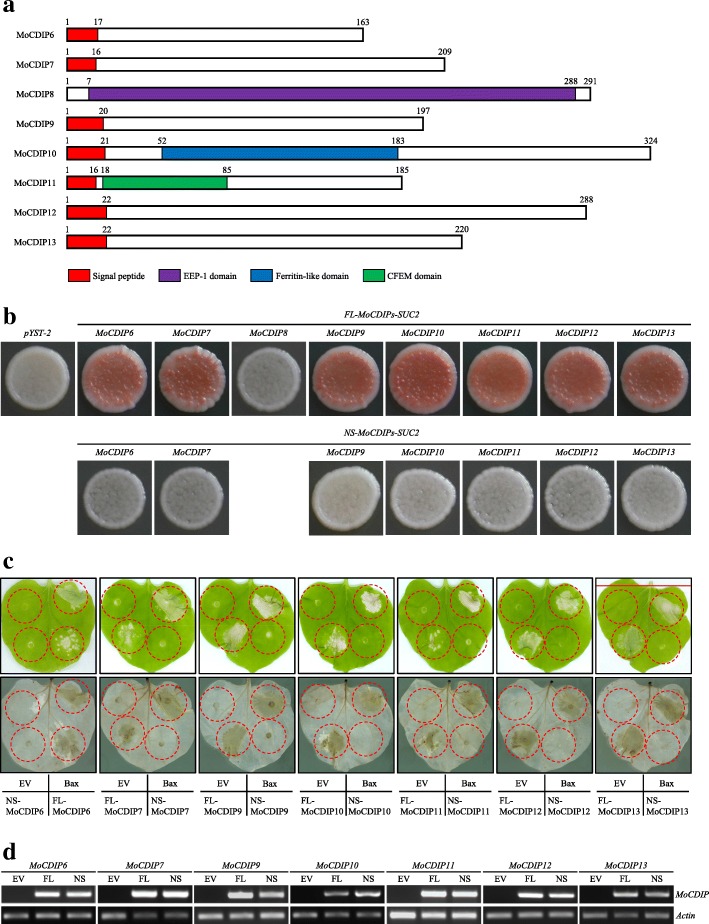
Sequence analysis and functional validation revealed that the signal peptides of seven MoCDIPs were required for cell death-inducing activity in *N. benthaminana*. **a** Structural analysis of the eight MoCDIPs. **b** Functional validation of the predicted signal peptides of seven MoCDIPs based on a yeast secretion assay system. *pYST-2*, yeast colony transformed with an empty vector containing a truncated yeast invertase *Suc2* gene lacking its original signal peptide sequence. *FL-MoCDIPs-Suc2*, yeast colonies transformed with full length *MoCDIPs* fused in frame with *Suc2*. *NS-MoCDIPs-Suc2*, truncated version lacking the predicted signal peptide sequence of *MoCDIPs* fused in-frame with *Suc2*. Yeast colonies grown on SD/−Leu medium were replica-plated onto sucrose selection medium for about 3 days. Strains unable to secrete invertase were inhibited on sucrose medium (white color), whereas strains able to secrete invertase could grow on sucrose medium (red color). **c** Transient expression of truncated non-signal peptide version of *MoCDIPs* did not cause cell death in *N. benthamiana* leaves. EV, an empty vector pGD. H_2_O_2_ accumulations in *N. benthamiana* leaves were shown by DAB staining. **d** RT-PCR analysis of *MoCDIPs* mRNA expression in agroinfiltrated *N. benthamiana* leaves. Total RNAs were extracted from infiltrated leaf tissues at 36 h post infiltration. EV, RT-PCR results from leaf tissues infiltrated with the empty vector pGD. FL, RT-PCR results from leaf tissues infiltrated with *FL-MoCDIPs*. NS, RT-PCR results from leaf tissues infiltrated with *NS-MoCDIPs*

The eight MoCDIP proteins, MoCDIP6 to MoCDIP13, are diverse in their sequences. Sequence analysis revealed that MoCDIP8 belonged to a family of endonuclease/exonuclease/phosphatase; MoCDIP10 was a putative ferritin-like family protein; and MoCDIP11 contained a conserved CFEM domain, which was unique to fungi (Kulkarni et al. 2003; Kou et al. 2017). As for MoCDIP6, MoCDIP7, MoCDIP9, MoCDIP12, and MoCDIP13, no putative conserved domains or motifs were identified (Fig. 3a). Unexpectedly, while MoCDIP6, MoCDIP7, MoCDIP9, MoCDIP10, MoCDIP11, MoCDIP12, and MoCDIP13 were predicted to contain a signal peptide, no signal peptide was predicted in MoCDIP8 based on SignalP 3.0 (http://www.cbs.dtu.dk/services/SignalP-3.0/) or SignalP 4.1 analysis (http://www.cbs.dtu.dk/services/SignalP-4.1/). A yeast secretion system was exploited to experimentally corroborate the predicted signal peptides. The sequences of full-length and truncated version lacking the predicted signal peptide sequence of *MoCDIPs* (referred to as *FL-MoCDIPs* and *NS-MoCDIPs*, respectively) were fused in frame with a truncated yeast invertase *Suc2* gene lacking its original signal peptide sequence. The fusion constructs were transformed into an invertase-deficient yeast mutant DBYα2445 (Lee et al. 2006). The transformed yeasts were plated on a sucrose selection medium. Consistent with the prediction, transformation of the fusion constructs of *FL-MoCDIP6-Suc2*, *FL-MoCDIP7-Suc2*, *FL-MoCDIP9-Suc2*, *FL-MoCDIP10-Suc2*, *FL-MoCDIP11-Suc2*, *FL-MoCDIP12-Suc2*, and *FL-MoCDIP13-Suc2* restored the secretion of invertase and resulted in yeast growth on sucrose (Fig. 3b). In contrast, the growth of yeasts transformed with a negative empty vector control pYST-2, as well as *FL-MoCDIP8-Suc2*, or *NS-MoCDIPs-Suc2* was inhibited on sucrose selection medium (Fig. 3b). These results confirmed the function of the predicted signal peptides to direct MoCDIP6, MoCDIP7, MoCDIP9, MoCDIP10, MoCDIP11, MoCDIP12, and MoCDIP13 to the secretory pathway.

*NS-MoCDIP6*, *NS-MoCDIP7*, *NS-MoCDIP9*, *NS-MoCDIP10*, *NS-MoCDIP11*, *NS-MoCDIP12*, and *NS-MoCDIP13* were also cloned into the expression vector pGD for agro-infiltration assay. The results showed that, while transient expression of *FL-MoCDIPs* repeatably induced cell death in *N. benthamiana* leaves, agro-infiltration of *NS-MoCDIPs* failed to induce cell death (Fig. 3c). Consistently, no H_2_O_2_ accumulation was observed in *N. benthamiana* leaves infiltrated with *Agrobacterium tumefaciens* carrying the *NS-MoCDIPs* constructs (Fig. 3c). RT-PCR analyses were carried out to examine the expression of *MoCDIPs* in the infiltrated *N. benthamiana* leaves, and the results showed that both the *FL-MoCDIPs* and *NS-MoCDIPs* were expressed at similar mRNA levels (Fig. 3d). Taken together, the results suggested that the signal peptides of MoCDIP6, MoCDIP7, MoCDIP9, MoCDIP10, MoCDIP11, MoCDIP12, and MoCDIP13 that led the secretion of the proteins to the extracellular space, were required for cell death inducing activity.

### *E. coli*-expressed recombinant MoCDIP6 and MoCDIP7 induced rice defense responses against blast fungus

**Fig. 4:**
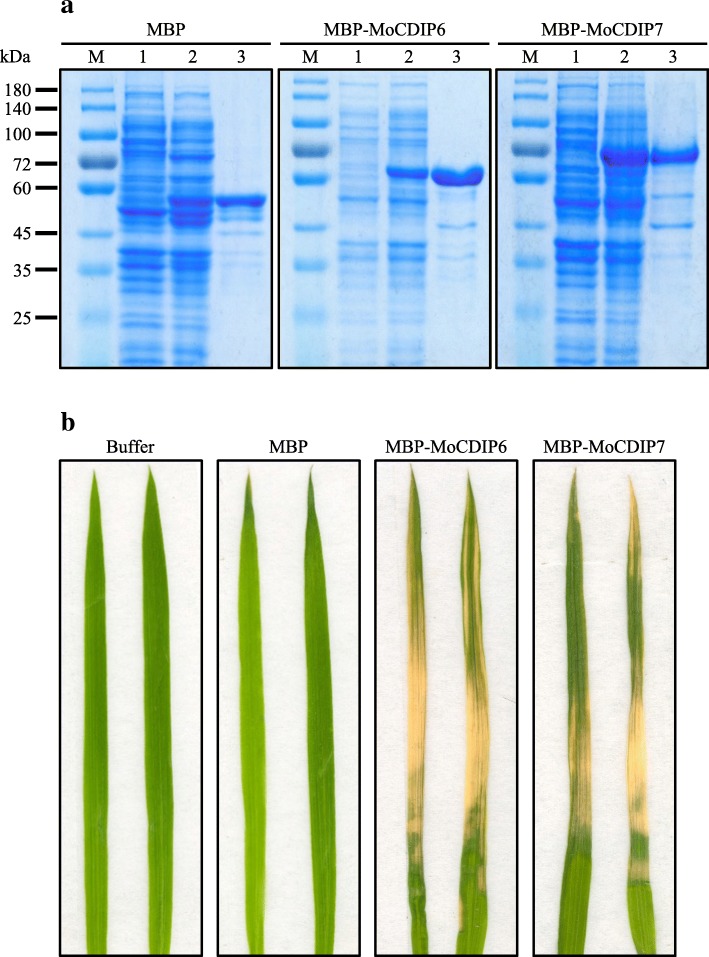
Recombinant MoCDIP6 and MoCDIP7 caused necrosis on leaves of rice seedlings. **a** Heterologous expression and purification of MBP, MBP-MoCDIP6, and MBP-MoCDIP7. M, protein molecular marker; 1, bacterial lysate before induction; 2, bacterial lysate after induction with IPTG; 3, purified MBP-tagged proteins. **b** Necrosis symptoms on leaves of Nipponbare rice seedlings sprayed with recombinant MoCDIP6 or MoCDIP7 (10 μM). The leaves were photographed 2 days after spray of the proteins

*NS-MoCDIP6*, *NS-MoCDIP7*, *NS-MoCDIP9*, *NS-MoCDIP10*, *NS-MoCDIP11*, *NS-MoCDIP12*, and *NS-MoCDIP13* were cloned into a pMAL-c2× vector downstream of a maltose-binding protein (MBP) tag. The resulting constructs were transformed into *E. coli* BL21 for protein expression. After IPTG-induction, all MBP-MoCDIP fusion proteins could be expressed in *E. coli* (data not shown). However, most of MBP-MoCDIPs formed inclusion bodies. At last, we successfully expressed and purified soluble recombinant MBP-MoCDIP6 and MBP-MoCDIP7 (Fig. 4a). The recombinant MoCDIP6 and MoCDIP7 solutions (10 μM) were sprayed onto rice seedlings. At two days after spray, rice seedlings displayed wilt and their leaves showed necrosis symptoms (Fig. 4b, Additional file 2: Figure S1). In contrast, rice seedlings treated with control buffer or MBP solution had no visible wilt or necrosis symptoms. These results confirmed cell death inducing activity of MoCDIP6 and MoCDIP7.

**Fig. 5:**
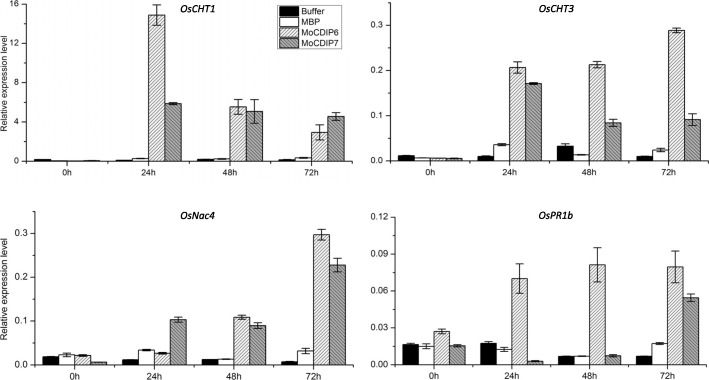
Recombinant MoCDIP6 and MoCDIP7 induced *PR* gene expression in rice seedlings. Nipponbare rice seedlings were sprayed with recombinant MoCDIP6 and MoCDIP7 (2 μM). Total RNA samples extracted from rice leaves at 0, 24, 48, or 72 hpi, were subjected to qRT-PCR

**Fig. 6:**
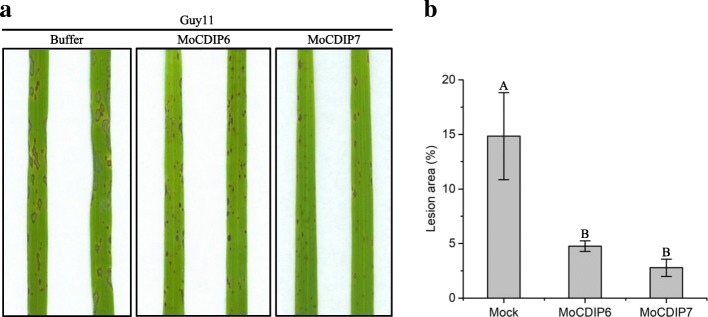
Pretreatment with recombinant MoCDIP6 or MoCDIP7 enhanced rice resistance against *M. oryzae*. **a** Disease symptoms on leaves of Nipponbare rice seedlings inoculated with a virulent *M. oryzae* isolate Guy11 with a 24-h pretreatment with recombinant MoCDIP 6 or MoCDIP7 (2 μM). **b** Disease lesion areas on leaves of MoCDIP6- or MoCDIP7-pretreated rice seedlings inoculated with Guy11. A and B indicate the significant differences according to LSD multiple range test at *P* ≤ 0.01

To determine whether MoCDIP6 and MoCDIP7 can elicit defense responses in rice, we sprayed the recombinant MoCDIP6 and MoCDIP7 solutions with a lower concentration (2 μM) onto rice seedlings, and evaluated the expression levels of four rice pathogenesis-related (*PR*) genes, *OsCht1*, *OsCht3*, *OsNac4* and *OsPR1b*. When sprayed with MoCIDP6 or MoCDIP7 at 2 μM, rice plants did not exhibit wilt or necrosis symptoms. Both MoCIDP6 and MoCDIP7 induced the expression of the four *PR* genes (Fig. 5). After 24 h pre-treatment with the lower dosage level of MoCIDP6 and MoCDIP7, the rice seedlings were further inoculated with the virulent fungal isolate Guy11. At 5 days post-inoculation (dpi), MoCIDP6- and MoCDIP7-pretreated plants, and control rice plants pretreated with the mock buffer all exhibited blast disease symptoms. However, while control plants exhibited typical and severe blast disease symptoms, most lesions observed on MoCIDP6- or MoCDIP7-pretreated plants were small and constrained (Fig. 6a). Consistently, disease lesion areas on leaves of MoCIDP6- or MoCDIP7-pretreated plants were significantly smaller than that on control plants (Fig. 6b). These results, therefore, demonstrated that MoCDIP6 and MoCDIP7 elicited defense responses in rice.

### *MoCDIPs* are dispensable for pathogenicity

To study whether *MoCDIPs* were required for pathogenicity of *M. oryzae*, we performed overexpression and disruption analyses of the eight *MoCDIP* genes. We successfully generated overexpression mutants (*OE*) for all eight *MoCDIP* genes and knock out mutants (*Δ*) for *MoCDIP6*, *MoCDIP7*, *MoCDIP8*, *MoCDIP10*, and *MoCDIP11* in Guy11 background (Table 1). The *OE*- or *Δ*-mutants and the wild type isolate Guy11 were characterized for their vegetative growth rate, sporulation, and pathogenicity. Compared with the wild type Guy11, *OE-MoCDIP6* showed a slower vegetative growth rate on oat medium, whereas the other *OE*- or *Δ*-mutants exhibited similar growth rate to that of Guy11 (Table 1). There was no significant difference in sporulation between the *OE*- or *Δ*-mutants and the wild type Guy11 (Table 1). The pathogenicity of the *OE*- or *Δ*-mutants was determined based on infection assays on detached barley leaves and on rice seedlings. Infection assays on detached barley leaves showed that the *OE*- or *Δ*-mutants developed similar disease lesions compared to that of Guy11. In addition, the *OE*- or *Δ*-mutants were highly virulent to rice cv. Nipponbare. No significant difference was observed for disease severity between the *OE*- or *Δ*-mutants and Guy11 (Table 1). These results suggested that the *MoCDIP* genes, at least *MoCDIP6*, *MoCDIP7*, *MoCDIP8*, *MoCDIP10*, and *MoCDIP11* were dispensable for pathogenicity of the rice blast fungus.

**Table 1:** Assays of growth, sporulation, and pathogenicity of the *MoCDIPs* overexpression and knock out mutants

Gene ID	Mutant	Vegetative growth (cm)	Sporulation (10^4^ spores·mL^− 1^)	Pathogenicity	
				Barley lesion (cm)^a^	Rice seedling (Disease level)^b^
	Guy11	6.59 ± 0.21	45.72 ± 6.48	6.71 ± 0.69	4–5
*MGG_01532*	*OE-MoCDIP6*	5.22 ± 0.08**	46.38 ± 7.36	6.25 ± 0.66	4–5
	*Δ-MoCDIP6*	6.60 ± 0.19	45.22 ± 8.50	7.05 ± 0.57	4–5
*MGG_03354*	*OE-MoCDIP7*	6.62 ± 0.14	46.60 ± 6.43	6.83 ± 0.58	4–5
	*Δ-MoCDIP7*	6.48 ± 0.17	39.22 ± 5.37	7.10 ± 0.70	4–5
*MGG_05038*	*OE-MoCDIP8*	6.59 ± 0.28	43.33 ± 4.97	6.13 ± 0.80	4–5
	*Δ-MoCDIP8*	6.83 ± 0.15	49.53 ± 6.62	6.42 ± 0.42	4–5
*MGG_08411*	*OE-MoCDIP9*	6.40 ± 0.07	47.23 ± 8.73	6.50 ± 0.48	4–5
	ND	ND	ND	ND	ND
*MGG_12275*	*OE-MoCDIP10*	6.55 ± 0.47	48.97 ± 6.89	6.88 ± 0.31	4–5
	*Δ-MoCDIP10*	6.65 ± 0.18	53.47 ± 7.80	6.63 ± 0.48	4–5
*MGG_12521*	*OE-MoCDIP11*	6.53 ± 0.19	43.10 ± 5.13	6.75 ± 0.50	4–5
	*Δ-MoCDIP11*	6.66 ± 0.13	51.58 ± 6.21	6.58 ± 0.30	4–5
*MGG_13283*	*OE-MoCDIP12*	6.51 ± 0.23	40.27 ± 6.90	6.21 ± 0.72	4–5
	ND	ND	ND	ND	ND
*MGG_14371*	*OE-MoCDIP13*	6.58 ± 0.09	39.72 ± 5.61	7.05 ± 0.50	4–5
	ND	ND	ND	ND	ND

^a^Infection on detached barley leaves was assessed by measuring the diameter of the lesions on the leaves;

^b^Disease severity was scored by following 0–5 scale (0–1: Resistant, 2: moderately resistant, 3: moderately susceptible, 4–5: severe susceptible);

Statistical significances (*P < 0.05 or **P < 0.01) between the *MoCDIPs* mutants and the wild type Guy11 were analyzed by student’s *t*-test; *ND* Not determined

## Discussion

Secreted effector proteins play critical roles in interactions between plants and phytopathogenic fungi (Giraldo and Valent 2013). The *M. oryzae* genome encodes a large number of secreted proteins (Dean et al. 2005; Yoshida et al. 2009; Choi et al. 2010). However, the function of majority of *M. oryzae* secreted proteins remain unknow. In our previous study, we performed transient expression assay of in planta-expressed *M. oryzae* secreted protein genes to screen cell death-inducing effector proteins (Chen et al. 2013). However, we had difficulty cloning a large number of in planta-expressed *M. oryzae* ORFs from cDNA generated from blast-infected rice leaves, mainly because the proportion of fungal RNA was relatively low (Chen et al. 2013). In the present study, we performed PCR or overlapping PCR using *M. oryzae* genomic DNA as template to clone those *M. oryzae* genes without introns or with only one intron. In addition, cDNA generated from mRNA of *M. oryzae* cultured under nitrogen starved conditions (Sharpee et al. 2017) was used as template for amplification of *M. oryzae* genes. We successfully cloned 98 more in planta-expressed *M. oryzae* putative secreted protein genes in total. The newly cloned genes, together with the 42 previously cloned genes (Chen et al. 2013), could also serve as resources for identifying *M. oryzae* secreted protein effectors with ability to suppress plant cell death (Sharpee et al. 2017), or having other different functions.

Studies from diverse fungal pathogens have shown that many fungal secreted protein effectors possessed abilities to induce plant cell death (Chen et al. 2013; Fang et al. 2016; Anderson et al. 2017). For example, using the agro-infiltration transient expression system, Fang et al. (2016) identified 13 cell death-inducing secreted proteins from the biotrophic fungus *Ustilaginoidea virens*; Anderson et al. (2017) identified cell death-inducing secreted proteins, including xylanase and inhibitor I9 domain containing proteins, from the necrotrophic fungal pathogen *Rhizoctonia solani*. In *M. oryzae*, several secreted proteins, including MoHrip1 (Chen et al. 2012), MoCDIP1 to MoCDIP5 (Chen et al. 2013), MoHrip2 (Chen et al. 2014), MSP1 (Wang et al. 2016), MoNLP1, MoNLP2 and MoNLP4 (Fang et al. 2017), and MoSM1 (Hong et al. 2017) had been demonstrated to possess cell death-inducing activity. In the present study, we identified eight novel in planta-expressed *M. oryzae* putative proteins, MoCDIP6 to MoCDIP13, that induced plant cell death. Seven out of the eight identified proteins, including MoCDIP6, MoCDIP7, MoCDIP9, MoCDIP10, MoCDIP11, MoCDIP12, and MoCDIP13 were predicted to contain a N-terminal signal peptide, and the function of the signal peptides for directing proteins to the secretory pathway was experimentally validated in yeast (Fig. 3b). Furthermore, truncation analysis revealed that the signal peptides were required for the seven proteins expressed in *N. benthamiana* leaves to induce cell death, suggesting that the secretion characteristics was required for cell death inducing activity of the seven MoCDIPs. Our results, together with those previously reported, demonstrated that secreted proteins from *M. oryzae* are enriched for cell death-inducing effectors.

Cell death plays a central role in plant-pathogen interactions (Kanneganti et al. 2006). As for hemibiotrophic and necrotrophic pathogens, some of cell death-inducing secreted protein effectors were believed to have function in facilitating the colonization during the necrotrophic phase (Sharpee et al. 2017). Indeed, many of these cell death-inducing protein effectors have been demonstrated to be required for pathogenicity, or contribute to the virulence of the pathogens (Ma et al. 2015; Qutob et al. 2006). On the other hand, some cell death-inducing proteins were shown to be dispensable for virulence of pathogens. For example, disruption of the *MSP1* gene (Wang et al. 2016), and the *MoNLP* family genes (Fang et al. 2017) in *M. oryzae* did not impair the fungal virulence on rice. In the present study, we also observed that overexpression of the eight identified *MoCDIP* genes, or targeted deletion of *MoCDIP6*, *MoCDIP7*, *MoCDIP8*, *MoCDIP10*, or *MoCDIP11* did not affect virulence of *M. oryzae* isolate Guy11 (Table 1). Since there were a number of cell death inducing secreted proteins in *M. oryzae*, one of the reasons for no virulence changes in the knock out mutants could be due to functional redundancy of closely related secreted proteins. Hence, further investigations would be needed to determine the biological roles of these cell death-inducing proteins in the interaction between *M. oryzae* and rice.

Many cell death-inducing proteins have been shown to elicit plant immune responses. Recent studies have revealed several cell death-inducing proteins that are recognized as PAMPs. For instance, multiple cytotoxic NLPs have been found to harbor a pattern of 20 amino acid residues (nlp20) that is recognized by Brassicaceae plant species (Böhm et al. 2014). The nlp20 motif interacts with the Arabidopsis LRR receptor protein RLP23, leading to PAMP-triggered immunity (PTI) transmitted via the RLP23-SOBIR1-BAK1 complex (Albert et al. 2015). XEG1, a *Phytophthora sojae* glycoside hydrolase 12 protein with cell death-inducing activity, and *Rhynchosporium commune* Cell Death Inducing 1 (RcCDI1) were also identified as PAMPs (Ma et al. 2015; Franco-Orozco et al. 2017). In *M. oryzae*, MoHrip1, MoHrip2, and MSP1 were demonstrated to be elicitors of defense responses in rice (Chen et al. 2012; Chen et al. 2014; Wang et al. 2016). In this study, we were able to express and purify soluble recombinant MoCDIP6 and MoCDIP7 from *E. coli*. We observed that application of ectopically expressed MoCDIP6 and MoCDIP7 induced the expression of *PR* genes in rice seedlings and increased the resistance of rice to *M. oryzae*, indicating that the two proteins triggered both cell death and immune responses in rice. It remains to be determined whether the other MoCDIPs can elicit plant immune responses and how plant cells recognize and respond to these proteins.

In summary, we identified eight novel *M. oryzae* cell death-inducing proteins, MoCDIP6 to MoCDIP13. Exogenous treatment of rice seedlings with recombinant MoCDIP6 and MoCDIP7 resulted in enhanced resistance to blast fungus, indicating that the two MoCDIP proteins triggered cell death and elicit defense responses in rice. The newly identified MoCDIP6 to MoCDIP13, together with previously identified MoCDIP1 to MoCDIP5, provide candidate targets for further studies to better understand the molecular mechanisms underlying the rice-*M. oryzae* interaction.

## Methods

### Plant materials and fungal isolates

Rice (*Oryza sativa*) materials used in this study were Nipponbare and MH3301. *M. oryzae* isolates used in this study included 70–15, Guy11 and 501–3.

### Cloning of *M. oryzae* genes encoding putative secreted proteins

*M. oryzae* genes encoding putative secreted proteins selected from the previously identified 851-gene list (Chen et al. 2013) were cloned by PCR amplification using gene-specific primers (Additional file 3: Table S2). Genomic DNA of *M. oryzae* isolate 70–15 was used as template for amplification of those *M. oryzae* genes without intron or with only one intron by PCR or overlapping PCR, and cDNA generated from mRNA of 70–15 cultured in minimal media lacking nitrogen was used as template for amplification of *M. oryzae* genes with introns. The amplified genes were cloned into a modified plant expression vector pGD (Goodin et al. 2002) that allowed for TA cloning of PCR products (Chen et al. 2009). All constructs were confirmed by sequencing.

### Agro-infiltration assays and DAB staining

The pGD-based *M. oryzae* gene constructs were transformed into *A. tumefaciens* strain GV3101 through electroporation. The *A. tumefaciens* clones containing *M. oryzae* gene constructs were cultured in liquid YEP media supplemented with rifampicin (50 μg·mL^− 1^) and kanamycin (50 μg·mL^− 1^). The *A. tumefaciens* cultures were collected by centrifugation, washed with sterile double-distilled H_2_O, and then resuspended in agro-infiltration buffer (10 mM MES, 10 mM MgCl_2_, and 150 μM actosyringone, pH 5.7) at an OD600 of 0.5 at room temperature for 2 h. Agro-infiltration experiments were carried out on leaves of 6-week-old *N. benthamiana* plants using needleless syringes*.*

For DAB staining, *N. benthamiana* leaves were incubated in DAB solution (1 mg·mL^− 1^) for 8 h at 25 °C in the dark. After staining, the leaves were washed with sterile double-distilled H_2_O, and were then boiled in bleaching solution containing ethanol and acetic acid (3:1) for 10–15 min.

### Gene expression analysis

Total RNA was extracted from *M. oryzae* mycelia, or rice leaves by using TRIzol reagent (Invitrogen Life Technologies, Carlsbad, CA) and treated with RNase-free DNase I (Ambion, Austin, TX). One microgram of total RNA was used for reverse transcription by using the Promega Reverse Transcription System (Promega, Madison, WI). Transcript levels of *MoCDIPs* and rice *PR* genes were determined by qRT-PCR under standard conditions with gene-specific primers (Additional file 3: Table S2). The experiments were performed with three replications.

### Yeast secretion assays

The pYST-2 vector (Lee et al. 2006), which contains a truncated invertase gene (*Suc2*) lacking the coding sequence of signal peptide, was used for validation of predicted signal peptides of the identified MoCDIPs. PCR fragments of *FL-MoCDIPs* and *NS-MoCDIPs* without the stop codon were amplified using specific primers (Additional file 3: Table S2). The fragments were cloned into pYST-2 using the restriction enzyme digestion and ligation method to fuse in-frame with *Suc2*. The resulting constructs were transformed into the invertase-deficient yeast mutant DBYα2445 (Lee et al. 2006). Transformants were cultured on SD/−Leu medium (1.7% yeast N base, 2% glucose, 0.5% ammonium sulfate, 0.069% complete amino acid supplement mixture minus leucine, and 2% agar), and yeast colonies were replica-plated onto sucrose selection medium (1% yeast extract, 2% peptone, 2% sucrose, and 2% agar) for secretion assays.

### Expression and purification of recombinant proteins

DNA fragments of *NS-MoCDIP6*, *NS-MoCDIP7*, *NS-MoCDIP9*, *NS-MoCDIP10*, *NS-MoCDIP11*, *NS-MoCDIP12*, or *NS-MoCDIP13* digested with *Bam*HI from the pGD-based gene constructs were inserted into the *Bam*HI site of the pMAL-c2× vector (New England Biolabs, Ipswich, USA), which harbors a MBP tag at the N-terminus of the fusion proteins. The resulting constructs were transformed into *E. coli* strain BL21. Expression of the recombinant MBP-NS-MoCDIPs were induced by 0.5 mM IPTG at 16 °C for overnight. MBP fusion proteins were affinity purified with amylose resin (New England Biolabs) according to manufacturer’s instructions. The recombinant proteins were evaluated by SDS-PAGE and staining with Coomassie Blue. The protein concentration was determined by using the Bradford protein assay kit (Bio-Rad Laboratories, Hercules, CA).

### Generation of overexpression and Knock-out mutants of *MoCDIPs*, and phenotype analyses

To overexpress the novel identified *MoCDIPs*, DNA fragments of *FL-MoCDIPs* digested with *Bam*HI from the pGD-based gene constructs were inserted into the *Bam*HI site of the pSM56 vector (Bourett et al. 2002), which contained a RP27 promoter for strong constitutive expression in *M. oryzae*. The resulting constructs were transformed into protoplasts of Guy11 through PEG-mediated transformation (Sweigard et al. 1992). Colonies growing on selection media with carbenicillin (50 μg·mL^− 1^) and hygromycin (150 μg·mL^− 1^) were evaluated by PCR to confirm the presence of the transgenes, and overexpression mutants were screened by qRT-PCR using specific primers (Additional file 3: Table S2).

To generate disruption mutants, genomic DNA fragments about 0.8 kb in length flanking the coding region of the targeted *MoCDIP* genes were amplified and were cloned into the pCX62 vector to flank the hygromycin phosphotransferase (*hph*) gene cassette. The resulting constructs were used for transformation of protoplasts of Guy11. Hygromycin-resistant colonies were screened by PCR for disruption of the *MoCDIP* genes.

Growth of fungal mycelia was tested by placing mycelium blocks (5 mm in diameter) on the center of a CM medium plate. The inoculated plates were incubated at 28 °C under dark conditions. The diameter of colonies was measured at 10 days. For quantification of sporulation, mycelium blocks (5 mm in diameter) were cultured on rice bran medium at 28 °C under dark conditions for 5 days, then were incubated at 28 °C under light for 3 days to induce spores. The plates were washed with 3 mL of distilled water. The conidial suspension was filtrated through a 100 μm mesh, and the number of spores was counted by hemocytometer.

### Plant inoculations

Infection assays on rice seedlings were performed in a greenhouse following a spraying inoculation method as previously described (Tian et al. 2016). Two-week-old rice seedlings were spray-inoculated with *M. oryzae* spores at a concentration of 2 × 10^5^ conidia·mL^− 1^. The inoculated seedlings were maintained under high humidity in the dark for 24 h, and were grown at 25 °C, under high humidity with a 16-h photoperiod for further 4–5 days.

Infection assays with detached barley leaves were conducted as previously described (Liu et al. 2011). Detached leaves of about eight-day-old barley seedlings were placed in plates with moist filter paper for drop inoculation. The plates were placed in dark at 25 °C for 24 h, and were then incubated at 25 °C with a 16-h photoperiod for further 4 days.

## Additional files

Additional file 1: **Table S1.** The 98 cloned in planta-expressed *M. oryzae* genes encoding putative secreted protein. (DOCX 34 kb)

Additional file 2: **Figure S1.** Recombinant MoCDIP6 and MoCDIP7 caused wilt symptoms on rice seedlings. (PPTX 7233 kb)

Additional file 3: **Table S2.** Primers used in this study. (DOCX 54 kb)

## Data Availability

The datasets supporting the conclusions of this article are included within the article and its additional files.

## Abbreviations

DAB: 3,3′-Diaminobenzidine tetrachloride
dpi: Days post-inoculation
hph: Hygromycin phosphotransferase
hpi: Hours post-inoculation
MBP: Maltose-binding protein
MoCDIPs: *M. oryzae* cell death–inducing proteins
PAMPs: Pathogen-associated molecular patterns
PR: Pathogenesis-related
PTI: PAMP-triggered immunity
qRT-PCR: Quantitative reverse-transcription PCR
