# Supplementary material for: Functional Identification of Novel Cell Death-inducing Effector Proteins from Magnaporthe oryzae
Source: Rice (N Y). 2019 Aug 6;12:59. doi: 10.1186/s12284-019-0312-z (PMC6684714; doi:10.1186/s12284-019-0312-z)
Supplement: Supplementary file 1 — Table S1. The 98 cloned in planta-expressed M. oryzae genes encoding putative secreted protein. (DOCX 34 kb) [file 12284_2019_312_MOESM1_ESM.docx]

**Additional file 1: Table S1** The 98 cloned *in-planta* expressed *M. oryzae* genes encoding putative secreted protein

| No. | Gene ID | | CDS size (bp) | | Annotation at Broad institute *M. oryzae* database | Comments |
| --- | --- | --- | --- | --- | --- | --- |
| 1 | *MGG_00081* | | 576 | | Hypothetical protein |  |
| 2 | *MGG_00083* | | 876 | | Hypothetical protein |  |
| 3 | *MGG_00148* | | 402 | | Hypothetical protein |  |
| 4 | *MGG_00210* | | 828 | | Hypothetical protein |  |
| 5 | *MGG_00230* | | 492 | | Hypothetical protein |  |
| 6 | *MGG_01403* | | 852 | | Fungal cellulose binding domain-containing protein |  |
| 7 | ***MGG_01532*** | | **492** | | **Hypothetical protein** | **Identified as MoCDIP6** |
| 8 | *MGG_01986* | | 987 | | Hypothetical protein |  |
| 9 | *MGG_01994* | | 809 | | Hypothetical protein |  |
| 10 | *MGG_02212* | | 498 | | Hypothetical protein |  |
| 11 | *MGG_02557* | | 966 | | Hypothetical protein |  |
| 12 | *MGG_02602* | | 330 | | Hypothetical protein |  |
| 13 | *MGG_03347* | | 801 | | Hypothetical protein |  |
| 14 | *MGG_03353* | | 702 | | Hypothetical protein |  |
| 15 | ***MGG_03354*** | | **634** | | **Hypothetical protein** | **Identified as MoCDIP7** |
| 16 | *MGG_04301* | | 438 | | Hypothetical protein |  |
| 17 | *MGG_04311* | | 1066 | | 3',5'-bisphosphate nucleotidase |  |
| 18 | *MGG_04343* | | 1026 | | Extradiol ring-cleavage dioxygenase |  |
| 19 | *MGG_04580* | | 381 | | Hypothetical protein |  |
| 20 | *MGG_04841* | | 726 | | Hypothetical protein |  |
| 21 | *MGG_04889* | | 579 | | Hypothetical protein |  |
| 22 | *MGG_04925* | | 597 | | Hypothetical protein |  |
| 23 | *MGG_04963* | | 175 | | Hypothetical protein |  |
| 24 | ***MGG_05038*** | | **1476** | | **EEP1 superfamily** | **Identified as MoCDIP8** |
| 25 | *MGG_05075* | | 663 | | Hypothetical protein |  |
| 26 | *MGG_05109* | | 1026 | | Hypothetical protein |  |
| 27 | *MGG_05518* | | 351 | | Hypothetical protein |  |
| 28 | *MGG_05896* | | 309 | | Hypothetical protein |  |
| 29 | *MGG_06302* | | 426 | | DNA polymerase epsilon subunit C |  |
| 30 | *MGG_06601* | | 312 | | Hypothetical protein |  |
| 31 | *MGG_06665* | | 378 | | Hypothetical protein |  |
| 32 | *MGG_06835* | | 762 | | Hypothetical protein |  |
| 33 | *MGG_06994* | | 270 | | Hypothetical protein |  |
| 34 | *MGG_07184* | | 339 | | Hypothetical protein |  |
| 35 | *MGG_07355* | | 447 | | Hypothetical protein |  |
| 36 | *MGG_07390* | | 897 | | Coagulation factor 5/8 type domain-containing protein |  |
| 37 | *MGG_07632* | | 930 | | Endonuclease/exonuclease/phosphatase |  |
| 38 | *MGG_07677* | | 780 | | Rhamnogalacturonan acetylesterase |  |
| 39 | | *MGG_07791* | | 416 | Surface protein 1 |  |
| 40 | | *MGG_07816* | | 486 | Hypothetical protein |  |

**Additional file 1: Table S1** The 98 cloned *in-planta* expressed *M. oryzae* genes encoding putative secreted protein (continued)

| No. | Gene ID | | CDS size (bp) | Annotation at Broad institute *M. oryzae* database | | | Comments |
| --- | --- | --- | --- | --- | --- | --- | --- |
| 41 | *MGG_07854* | | 687 | | Hypothetical protein |  | |
| 42 | *MGG_07869* | | 390 | | Hypothetical protein |  | |
| 43 | *MGG_07972* | | 876 | | Hypothetical protein |  | |
| 44 | *MGG_08024* | | 309 | | Hypothetical protein |  | |
| 45 | *MGG_08230* | | 407 | | Hypothetical protein |  | |
| 46 | *MGG_08300* | | 498 | | Hypothetical protein |  | |
| 47 | *MGG_08334* | | 879 | | Hypothetical protein |  | |
| 48 | ***MGG_08411*** | | **594** | | **Hypothetical protein** | **Identified as MoCDIP9** | |
| 49 | *MGG_08428* | | 336 | | Hypothetical protein |  | |
| 50 | *MGG_08469* | | 417 | | Hypothetical protein |  | |
| 51 | *MGG_08480* | | 876 | | Alpha/beta hydrolase |  | |
| 52 | *MGG_08644* | | 588 | | DNase1 protein |  | |
| 53 | *MGG_08715* | | 414 | | Hypothetical protein |  | |
| 54 | *MGG_08944* | | 429 | | Hypothetical protein |  | |
| 55 | *MGG_09095* | | 978 | | Alpha-L-arabinofuranosidase axhA-2 |  | |
| 56 | *MGG_09147* | | 1047 | | Hypothetical protein |  | |
| 57 | *MGG_09268* | | 1050 | | Hypothetical protein |  | |
| 58 | *MGG_09374* | | 539 | | Hypothetical protein |  | |
| 59 | *MGG_09379* | | 366 | | Hypothetical protein |  | |
| 60 | *MGG_09420* | | 903 | | Hypothetical protein |  | |
| 61 | *MGG_09629* | | 570 | | Hypothetical protein |  | |
| 62 | *MGG_09657* | | 330 | | Hypothetical protein |  | |
| 63 | *MGG_09742* | | 807 | | Hypothetical protein |  | |
| 64 | *MGG_09826* | | 420 | | Hypothetical protein |  | |
| 65 | *MGG_09848* | | 555 | | Hypothetical protein |  | |
| 66 | *MGG_10024* | | 566 | | Hypothetical protein |  | |
| 67 | *MGG_10065* | | 507 | | Hypothetical protein |  | |
| 68 | *MGG_10080* | | 744 | | Hypothetical protein |  | |
| 69 | *MGG_10206* | | 340 | | Hypothetical protein |  | |
| 70 | *MGG_10237* | | 774 | | Hypothetical protein |  | |
| 71 | *MGG_10244* | | 384 | | Hypothetical protein |  | |
| 72 | *MGG_10276* | | 597 | | Hypothetical protein |  | |
| 73 | *MGG_10456* | | 454 | | Hypothetical protein |  | |
| 74 | *MGG_10926* | | 615 | | Hypothetical protein |  | |
| 75 | *MGG_11072* | | 381 | | Hypothetical protein |  | |
| 76 | *MGG_11224* | | 908 | | Hypothetical protein |  | |
| 77 | *MGG_11304* | 537 | | | Hypothetical protein |  | |
| 78 | *MGG_11606* | 1029 | | | Hypothetical protein |  | |
| 79 | *MGG_11627* | 363 | | | Hypothetical protein |  | |
| 80 | ***MGG_12275*** | **975** | | | **Ferritin-like superfamily** | **Identified as MoCDIP10** | |

**Additional file 1: Table S1** The 98 cloned *in-planta* expressed *M. oryzae* genes encoding putative secreted protein (continued)

| No. | Gene ID | CDS size (bp) | Annotation at Broad institute *M. oryzae* database | Comments |
| --- | --- | --- | --- | --- |
| 81 | *MGG_12313* | 897 | Hypothetical protein |  |
| 82 | ***MGG_12521*** | **558** | **CFEM superfamily** | **Identified as MoCDIP11** |
| 83 | *MGG_12847* | 695 | Hypothetical protein |  |
| 84 | *MGG_12858* | 762 | Hypothetical protein |  |
| 85 | *MGG_13063* | 972 | Hypothetical protein |  |
| 86 | ***MGG_13283*** | **867** | **Hypothetical protein** | **Identified as MoCDIP12** |
| 87 | *MGG_13325* | 456 | Hypothetical protein |  |
| 88 | *MGG_13863* | 438 | Hypothetical protein |  |
| 89 | *MGG_13872* | 486 | Hypothetical protein |  |
| 90 | ***MGG_14371*** | **663** | **Hypothetical protein** | **Identified as MoCDIP13** |
| 91 | *MGG_14652* | 441 | Hypothetical protein |  |
| 92 | *MGG_14725* | 402 | Hypothetical protein |  |
| 93 | *MGG_14965* | 627 | Hypothetical protein |  |
| 94 | *MGG_15022* | 432 | Hypothetical protein |  |
| 95 | *MGG_15106* | 450 | Hypothetical protein |  |
| 96 | *MGG_15371* | 627 | Hypothetical protein |  |
| 97 | *MGG_15374* | 444 | Hypothetical protein |  |
| 98 | *MGG_15443* | 348 | Hypothetical protein |  |
