# Supplementary material for: Functional Identification of Novel Cell Death-inducing Effector Proteins from Magnaporthe oryzae
Source: Rice (N Y). 2019 Aug 6;12:59. doi: 10.1186/s12284-019-0312-z (PMC6684714; doi:10.1186/s12284-019-0312-z)
Supplement: Supplementary file 2 — Figure S1. Recombinant MoCDIP6 and MoCDIP7 caused wilt symptoms on rice seedlings. (PPTX 7233 kb) [file 12284_2019_312_MOESM2_ESM.pptx]

## Slide 1
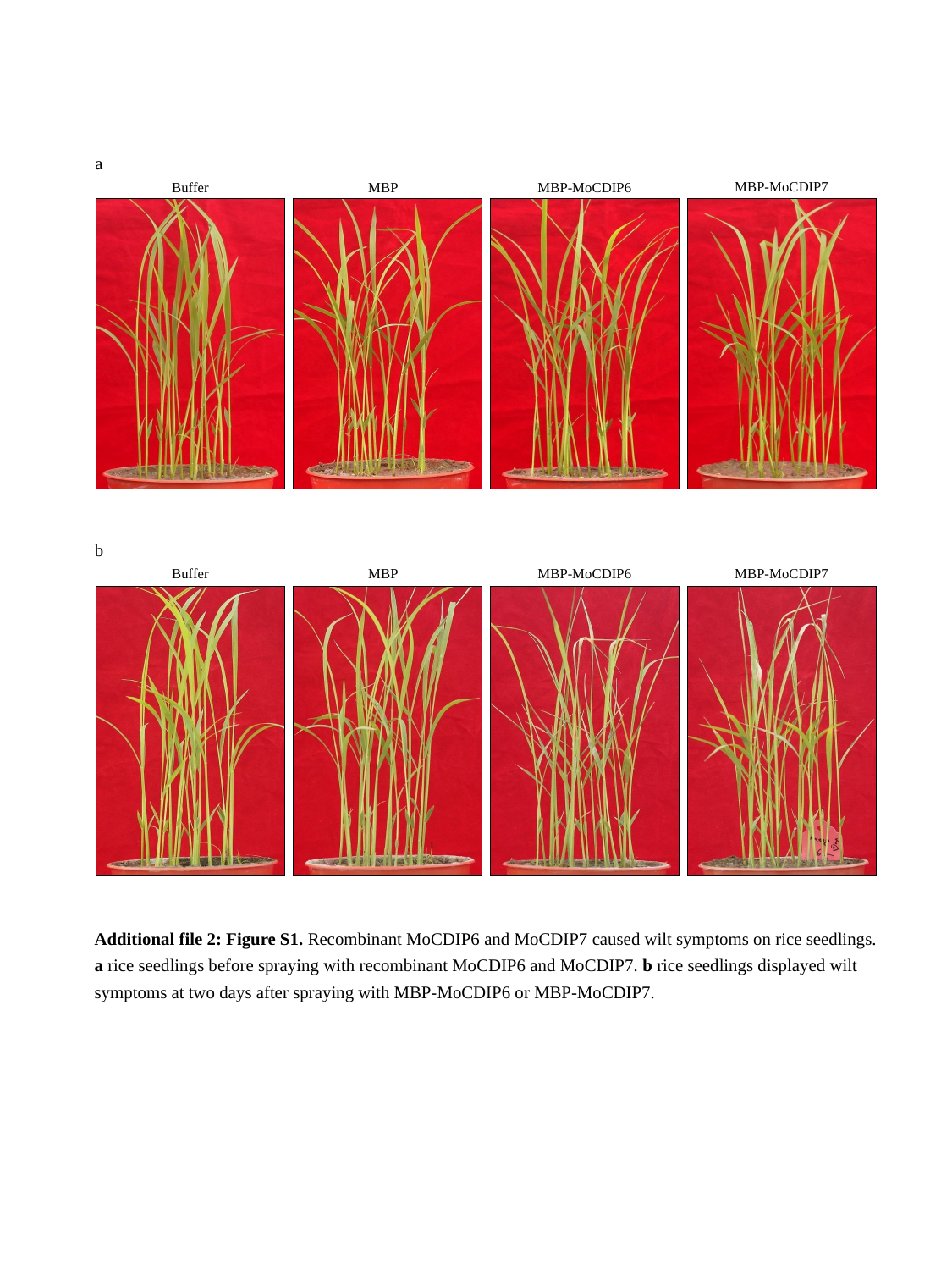

a
MBP-MoCDIP7
Buffer
MBP
MBP-MoCDIP6
b
MBP-MoCDIP7
Buffer
MBP
MBP-MoCDIP6
Additional file 2: Figure S1. Recombinant MoCDIP6 and MoCDIP7 caused wilt symptoms on rice seedlings. a rice seedlings before spraying with recombinant MoCDIP6 and MoCDIP7. b rice seedlings displayed wilt symptoms at two days after spraying with MBP-MoCDIP6 or MBP-MoCDIP7.
