# Supplementary material for: Functional Identification of Novel Cell Death-inducing Effector Proteins from Magnaporthe oryzae
Source: Rice (N Y). 2019 Aug 6;12:59. doi: 10.1186/s12284-019-0312-z (PMC6684714; doi:10.1186/s12284-019-0312-z)
Supplement: Supplementary file 3 — Table S2. Primers used in this study. (DOCX 54 kb) [file 12284_2019_312_MOESM3_ESM.docx]

**Additional file 3: Table S2** Primers used in this study

| **No.** | **Primers** | **Sequence: 5′- 3′** | **Comments** |
| --- | --- | --- | --- |
| 1 | 00081-F | AATGCAGTTCACCGTCGCAT | F primer to amplify *MGG_00081* |
| 2 | 00081-R | CCAGTTTACTCCTGGCTGTAGAC | R primer to amplify *MGG_00081* |
| 3 | 00083-F | TATGAGATTCGAGACTCTGATCGT | F primer to amplify *MGG_00083* |
| 4 | 00083-R | TATGAAGATCCAGAACCTGTCGG | R primer to amplify *MGG_00083* |
| 5 | 00148-F | TATGAAGATCCAGAACCTGTCGG | F primer to amplify *MGG_00148* |
| 6 | 00148-R | GTTATATTTCGGCTTCCCTGC | R primer to amplify *MGG_00148* |
| 7 | 00210-F | TATGCGCCGTCCCGCACTCC | F primer to amplify *MGG_00210* |
| 8 | 00210-R | GGGGCTATAATAAGTCTAAAATTGTAGG | R primer to amplify *MGG_00210* |
| 9 | 00230-F | TATGCAATACCAGATTCTGAACG | F primer to amplify *MGG_00230* |
| 10 | 00230-R | CTTAGCTGAGCTTGGTCTTCTGC | R primer to amplify *MGG_00230* |
| 11 | 01403-F | TATGATGTCTCAGACACGCATCCT | F primer to amplify *MGG_01403* |
| 12 | 01403-R | ATCACTGCGGCTGGAACTGGT | R primer to amplify *MGG_01403* |
| 13 | 01532-F | TATGAAGTTCTCCGCCATCA | F primer to amplify *MGG_01532* |
| 14 | 01532-NS-F | ATGGCTCCCACCAGCACCCCT | F primer to amplify *NS*-*MGG_01532* |
| 15 | 01532-R | CTCACAGGATGAAACCCCTCTG | R primer to amplify *MGG_01532* |
| 16 | 01986-F | TATGCCTTGCTCTTCCATTTTCTT | F primer to amplify *MGG_01986* |
| 17 | 01986-R | CTCAGATCCTCATCATCCTGCTG | R primer to amplify *MGG_01986* |
| 18 | 01994-F | TATGTCGACTGCCCAAACAATCC | F primer to amplify *MGG_01994* |
| 19 | 01994-R | TCACCCTAGTATGTAACCGTCACCA | R primer to amplify *MGG_01994* |
| 20 | 02212-F | TATGCGTCTCTCAACCTTCCTA | F primer to amplify *MGG_02212* |
| 21 | 02212-R | TCTACGAGGAGGCAGCGATAG | R primer to amplify *MGG_02212* |
| 22 | 02557-F | GATGCAAATCACAAAGATTGCCG | F primer to amplify *MGG_02557* |
| 23 | 02557-R | GTCATTTCTTGCCTTTGCCTCC | R primer to amplify *MGG_02557* |
| 24 | 02602-F | CACAATGCAGTTCAAGTCGATAGC | F primer to amplify *MGG_02602* |
| 25 | 02602-R | TCTTACAGCAACATGGCAGCG | R primer to amplify *MGG_02602* |
| 26 | 03347-F | CATGGCGGCCACAGCGTC | F primer to amplify *MGG_03347* |
| 27 | 03347-R | CCTCAAGACCGGCTGGGAATC | R primer to amplify *MGG_03347* |
| 28 | 03353-F | CATGGTCTCGATGATCAAGCAATC | F primer to amplify *MGG_03353* |
| 29 | 03353-R | TCTAAGTAGTCGGCTGCACTGGAAT | R primer to amplify *MGG_03353* |
| 30 | 03354-F | TATGCGCTCTGCCATGATCA | F primer to amplify *MGG_03354* |
| 31 | 03354-NS-F | ATGCACCCTCCCAAGCCGT | F primer to amplify *NS-MGG_03354* |
| 32 | 03354-R | CGATCTACAAGAAAGCAACGG | R primer to amplify *MGG_03354* |
| 33 | 04301-F | CATGAAATGCAACAACATCATCCT | F primer to amplify *MGG_04301* |
| 34 | 04301-R | CTTACATAATATTGCAGCCCTCTTCT | R primer to amplify *MGG_04301* |
| 35 | 04311-F | GATGGCCACCTACGCCAAG | F primer to amplify *MGG_04311* |
| 36 | 04311-R | CGCTAGTTCTTCCCCAAAACCTC | R primer to amplify *MGG_04311* |
| 37 | 04343-F | AATGTCCCGCCGCGCAAC | F primer to amplify *MGG_04343* |
| 38 | 04343-R | GTCAGTCCCAATAGTACGAATGAAT | R primer to amplify *MGG_04343* |
| 39 | 04580-F | TATGCACCTGTCCACAGTCTCGC | F primer to amplify *MGG_04580* |
| 40 | 04580-R | GTCAGCCACCCTTGTTTCTAAACT | R primer to amplify *MGG_04580* |
| 41 | 04841-F | GATGCGTTCCTCATCCATCATC | F primer to amplify *MGG_04841* |
| 42 | 04841-R | GTCATGCAAGCAGGTAGACGAG | R primer to amplify *MGG_04841* |
| 43 | 04889-F | TATGCGTTTCTCTACCGTGGC | F primer to amplify *MGG_04889* |
| 44 | 04889-R | TCTATTTGCAAGTAAGGGTGATGG | R primer to amplify *MGG_04889* |
| 45 | 04925-F | AATGGGTTCCATCACGATGATAC | F primer to amplify *MGG_04925* |
| 46 | 04925-R | CTCAAATGCCATATCCATAACCTAAAC | R primer to amplify *MGG_04925* |
| 47 | 04963-F | GGCAAGATGGTTAAACAAAACG | F primer to amplify *MGG_04963* |
| 48 | 04963-R | GTCATTGATCGTCGTCGACTAAT | R primer to amplify *MGG_04963* |
| 49 | 05038-F | TTATGGCCTCCCTCCCCCTC | F primer to amplify *MGG_05038* |
| 50 | 05038-R | TTCATCGAAGAATCACGTCACCAAC | R primer to amplify *MGG_05038* |
| 51 | 05075-F | AATGCACGGCATCACCATCTC | F primer to amplify *MGG_05075* |
| 52 | 05075-R | TCTAGTCGCTCGGCTTCAGTGTT | R primer to amplify *MGG_05075* |
| 53 | 05109-F | GATGGACATAGCAGTGCCGC | F primer to amplify *MGG_05109* |
| 54 | 05109-R | TTTACCATGGCCTTATGCTGTCT | R primer to amplify *MGG_05109* |
| 55 | 05518-F | CATGCATATAATCCACATTTCCAAGT | F primer to amplify *MGG_05518* |
| 56 | 05518-R | GTCAGCTATCGAATCTACGTTCATC | R primer to amplify *MGG_05518* |
| 57 | 05896-F | TATGAAGCTCGCCACCGGTT | F primer to amplify *MGG_05896* |
| 58 | 05896-R | ACTAGCGGCTTAACTGGCAAGTGT | R primer to amplify *MGG_05896* |
| 59 | 06302-F | AATGCCGCCAAGAAGATCAG | F primer to amplify *MGG_06302* |
| 60 | 06302-R | CACTAGGTGACCTTCTTTTTCCGT | R primer to amplify *MGG_06302* |
| 61 | 06601-F | GGATGGTCTTTATAACGCGCCT | F primer to amplify *MGG_06601* |
| 62 | 06601-R | ATCACCTCGCACCACCAGTGAG | R primer to amplify *MGG_06601* |
| 63 | 06665-F | CTGGATGCAATTCTCTACACTCCTTT | F primer to amplify *MGG_06665* |
| 64 | 06665-R | ATCACGGCCTGCCATTGCC | R primer to amplify *MGG_06665* |
| 65 | 06835-F | CGATGATTTCCTCCAGGATCACC | F primer to amplify *MGG_06835* |
| 66 | 06835-R | AACTATCCCCTGCGGGCGT | R primer to amplify *MGG_06835* |
| 67 | 06994-F | ACTTATGGCCAGCCCTGCG | F primer to amplify *MGG_06994* |
| 68 | 06994-R | CTTATCTTGCCGCTGGCGGC | R primer to amplify *MGG_06994* |
| 69 | 07184-F | TATGCATCTTTCATCCCTTTTC | F primer to amplify *MGG_07184* |
| 70 | 07184-R | ACTACTCCTGCAACTCGAGACC | R primer to amplify *MGG_07184* |
| 71 | 07355-F | AATGAAAACCCAACGCGTCAC | F primer to amplify *MGG_07355* |
| 72 | 07355-R | ATCAACCGCCAAAGCCAAAT | R primer to amplify *MGG_07355* |
| 73 | 07390-F | GATGTTGTTTACGCCGATTCGAT | F primer to amplify *MGG_07390* |
| 74 | 07390-R | CTTACTGCCCAAACACGCTTGT | R primer to amplify *MGG_07390* |
| 75 | 07632-F | GATGCTTCCCACCAACCTCAT | F primer to amplify *MGG_07632* |
| 76 | 07632-R | CCTCATTTCATTGTGATATCCGC | R primer to amplify *MGG_07632* |
| 77 | 07677-F | CAATGCAGCTCCCCACGTC | F primer to amplify *MGG_07677* |
| 78 | 07677-R | GTTAGTACACCCGTGGGTAGTTGG | R primer to amplify *MGG_07677* |
| 79 | 07791-F | GATGCAGTTCTCAACCACCACC | F primer to amplify *MGG_07791* |
| 80 | 07791-R | AATAACCCCCTCACAGAGCCTG | R primer to amplify *MGG_07791* |
| 81 | 07816-F | TATGGCACCCTCACAACCACT | F primer to amplify *MGG_07816* |
| 82 | 07816-R | TTCAGTACCGACTGTGGCTCC | R primer to amplify *MGG_07816* |
| 83 | 07854-F | GATGCACATTTCCAAAGCCTCC | F primer to amplify *MGG_07854* |
| 84 | 07854-R | ATCACTGGTGTTGGCCCTTCG | R primer to amplify *MGG_07854* |
| 85 | 07869-F | CATGCATTCTTCCAACATTCTCC | F primer to amplify *MGG_07869* |
| 86 | 07869-R | ACTATATAGCCTCCTTCGCAGCC | R primer to amplify *MGG_07869* |
| 87 | 07972-F | GATGTACATTTTTAACCTTCCCAG | F primer to amplify *MGG_07972* |
| 88 | 07972-R | ACTACATTCCAGGCTGTAGCCC | R primer to amplify *MGG_07972* |
| 89 | 08024-F | GTATGATCTTCAACGTTTTTACACTGC | F primer to amplify *MGG_08024* |
| 90 | 08024-R | CTTTACTGCTTGGGAGGAGGG | R primer to amplify *MGG_08024* |
| 91 | 08230-F | CAATATGCAGATCTTCAAGATTGTT | F primer to amplify *MGG_08230* |
| 92 | 08230-R | TTTAGCGATCATCACGAACTGT | R primer to amplify *MGG_08230* |
| 93 | 08300-F | TTATGCAGTTCTCCATCTACGC | F primer to amplify *MGG_08300* |
| 94 | 08300-R | TCTATGCCGTGCTGGCAGT | R primer to amplify *MGG_08300* |
| 95 | 08334-F | GATGGTTCTCATAACGCAAATTCT | F primer to amplify *MGG_08334* |
| 96 | 08334-R | CTTAGTGGTAGTAACCTCTTAGCTTC | R primer to amplify *MGG_08334* |
| 97 | 08411-F | TATGCGCATCACCAGCCG | F primer to amplify *MGG_08411* |
| 98 | 08411-NS-F | ATGATGCCGACCGGCTTGCT | F primer to amplify *NS*-*MGG_08411* |
| 99 | 08411-R | CTTAGTCCTTCTTCTGGGTTTCCTT | R primer to amplify *MGG_08411* |
| 100 | 08428-F | AATGCAGCTTCAGTCAATCATCG | F primer to amplify *MGG_08428* |
| 101 | 08428-R | CTCAAAATTTAGGATCGTGGCTC | R primer to amplify *MGG_08428* |
| 102 | 08469-F | GATGGTGCGCGCAAAGAT | F primer to amplify *MGG_08469* |
| 103 | 08469-R | CTTATAGTTCATGCTTTTTGCCCAC | R primer to amplify *MGG_08469* |
| 104 | 08480-F | TATGGTCTCCTTCACCACCCTC | F primer to amplify *MGG_08480* |
| 105 | 08480-R | TTTACGCCTTCAAGAAAGCCTC | R primer to amplify *MGG_08480* |
| 106 | 08644-F | TATGCCATCTCCACGCTGCCT | F primer to amplify *MGG_08644* |
| 107 | 08644-R | ATTACGAATCCCCTAGCCCCTCTC | R primer to amplify *MGG_08644* |
| 108 | 08715-F | TATGCGCGTTTCCATTTTCATC | F primer to amplify *MGG_08715* |
| 109 | 08715-R | TCTAGTATCTCCCGTACCTGTGGCT | R primer to amplify *MGG_08715* |
| 110 | 08944-F | GATGCACTTCACCACCGTCG | F primer to amplify *MGG_08944* |
| 111 | 08944-R | TTCAAGCCTGGGCCTTGG | R primer to amplify *MGG_08944* |
| 112 | 09095-F | ATTATGCGCTTCTTCGAGACC | F primer to amplify *MGG_09095* |
| 113 | 09095-R | TCTAACGACGGAGCGTCAGC | R primer to amplify *MGG_09095* |
| 114 | 09147-F | GATGCTTGTCCGTGCGATTGTAT | F primer to amplify *MGG_09147* |
| 115 | 09147-R | GCTATGCACCGTATGCCTGCTT | R primer to amplify *MGG_09147* |
| 116 | 09268-F | TATGCATCAACGACAGGGC | F primer to amplify *MGG_09268* |
| 117 | 09268-R | GTTATCTACGATCTTGGTACGGC | R primer to amplify *MGG_09268* |
| 118 | 09347-F | AATGTACGTCCCGCGGGAGC | F primer to amplify *MGG_09347* |
| 119 | 09347-R | CTCACCACCCGCCACCCTG | R primer to amplify *MGG_09347* |
| 120 | 09379-F | TATGCGGTCCCAAGCCCTC | F primer to amplify *MGG_09379* |
| 121 | 09379-R | CCTAAGTGCTTTTAACCTGGTCCC | R primer to amplify *MGG_09379* |
| 122 | 09420-F | GATGCGTCTAATCCTCATTTTC | F primer to amplify *MGG_09420* |
| 123 | 09420-R | CTCACCACAGAGTGACTATAAACG | R primer to amplify *MGG_09420* |
| 124 | 09629-F | TATGCATCCCTCGTCGTTTCTC | F primer to amplify *MGG_09629* |
| 125 | 09629-R | CCTATGCGGTAAACGTCCTTGTC | R primer to amplify *MGG_09629* |
| 126 | 09657-F | AATGCAGATCTCCCACATTGCC | F primer to amplify *MGG_09657* |
| 127 | 09657-R | ACTACTTCTGCTCCTGGTTGCCAC | R primer to amplify *MGG_09657* |
| 128 | 09742-F | TATGCAGCTCCTCAAAGGTCTCG | F primer to amplify *MGG_09742* |
| 129 | 09742-R | ATCACGCAGGTCCACCCGC | R primer to amplify *MGG_09742* |
| 130 | 09826-F | GATGCATGCCTACAAAGCCTG | F primer to amplify *MGG_09826* |
| 131 | 09826-R | TTCAAAACTCCTTGCACCTAAACTC | R primer to amplify *MGG_09826* |
| 132 | 09848-F | TATGCAGTTCAAGGCCATCCT | F primer to amplify *MGG_09848* |
| 133 | 09848-R | GTTATTTGCAGGTGAGGGACTTG | R primer to amplify *MGG_09848* |
| 134 | 10024-F | CTATGAAGTCCAGCGCAGTAATCC | F primer to amplify *MGG_10024* |
| 135 | 10024-R | CTCTAGCTCGCCGTACCCGTA | R primer to amplify *MGG_10024* |
| 136 | 10065-F | AATGCAGATTCTCAAGATCACTTGG | F primer to amplify *MGG_10065* |
| 137 | 10065-R | GTTACTCAGATTCTCCGGGGG | R primer to amplify *MGG_10065* |
| 138 | 10080-F | AATGCGCGTCTCCAGCTGGT | F primer to amplify *MGG_10080* |
| 139 | 10080-R | ACTAGGCGACTTGGAGCTTTCTGTT | R primer to amplify *MGG_10080* |
| 140 | 10206-F | TATGCCTCGCCTCTTCCTCC | F primer to amplify *MGG_10206* |
| 141 | 10206-R | GCTAACGGCCAACCACGAG | R primer to amplify *MGG_10206* |
| 142 | 10237-F | GATGAAGCTCACCCAAGCC | F primer to amplify *MGG_10237* |
| 143 | 10237-R | TGTTAGTAAGAGTCTTCCATATCGG | R primer to amplify *MGG_10237* |
| 144 | 10244-F | AATGAAGTCGGCAATCATCCTCG | F primer to amplify *MGG_10244* |
| 145 | 10244-R | ATTAGGCCTTGGTCATCTTGTCCC | R primer to amplify *MGG_10244* |
| 146 | 10276-F | TATGCAGCTTTCAAACTTTCTTTCG | F primer to amplify *MGG_10276* |
| 147 | 10276-R | ATCAGAGCTGGACCGGGTCG | R primer to amplify *MGG_10276* |
| 148 | 10456-F | TATGCAGTTCATCTCTACCTTCCTCG | F primer to amplify *MGG_10456* |
| 149 | 10456-R | CCCTTACTTCCTGTCGCACTCA | R primer to amplify *MGG_10456* |
| 150 | 10926-F | AATGTCAGGCTTCACGGGC | F primer to amplify *MGG_10926* |
| 151 | 10926-R | GTTACATCTCCCTCTGAATTTTCA | R primer to amplify *MGG_10926* |
| 152 | 11072-F | TATGCAGATCAAGACTTTCGCC | F primer to amplify *MGG_11072* |
| 153 | 11072-R | CTTAGTAGGTGCAAGTGCACTCG | R primer to amplify *MGG_11072* |
| 154 | 11224-F | GATGCGATCCACGACTATTCTCTC | F primer to amplify *MGG_11224* |
| 155 | 11224-R | TCTTAGGCGATGGTCCACTTGA | R primer to amplify *MGG_11224* |
| 156 | 11304-F | TATGCAAATCTCCAACGCGTTT | F primer to amplify *MGG_11304* |
| 157 | 11304-R | ACTAGGCATCTCGCATAGCTGTAGG | R primer to amplify *MGG_11304* |
| 158 | 11606-F | TATGGCGCAATTCAGGAAG | F primer to amplify *MGG_11606* |
| 159 | 11606-R | GCTAGCACGCAGTAACAGTTG | R primer to amplify *MGG_11606* |
| 160 | 11627-F | AATGGGAGACCAGAAAGTCATGG | F primer to amplify *MGG_11627* |
| 161 | 11627-R | CCTACTTCTCAGGATGCTCGTCTG | R primer to amplify *MGG_11627* |
| 162 | 12275-F | AATGCCTTCCATCATCAAGACAG | F primer to amplify *MGG_11275* |
| 163 | 12275-NS-F | ATGCTCCCCTCACAGTCGAG | F primer to amplify *NS*-*MGG_11275* |
| 164 | 12275-R | TTTAGAGAGAGCCAGGAGCCAAGTT | R primer to amplify *MGG_11275* |
| 165 | 12313-F | AATGAGGCATCGATTCTCATTAACC | F primer to amplify *MGG_11313* |
| 166 | 12313-R | CTTACATAATTAGACCTGCCACTCCTACC | R primer to amplify *MGG_11313* |
| 167 | 12521-F | AATGAAGTTCTCCATCATCCTCG | F primer to amplify *MGG_11521* |
| 168 | 12521-NS-F | ATGCAGGACCTCTCTTCCCT | F primer to amplify *NS*-*MGG_11521* |
| 169 | 12521-R | CTTACAAAGCAAGGACGGCAC | R primer to amplify *MGG_11521* |
| 170 | 12847-F | AATGCGCTTCACTGCTGCTGC | F primer to amplify *MGG_12847* |
| 171 | 12847-R | GTTAAGCCAGGAGGGCAAGGG | R primer to amplify *MGG_12847* |
| 172 | 12858-F | TATGAGGCAATATGCAACCC | F primer to amplify *MGG_12858* |
| 173 | 12858-R | CTTACAACACTGCGGCTAAAG | R primer to amplify *MGG_12858* |
| 174 | 13063-F | AATGCGCGTCTCAACCATCG | F primer to amplify *MGG_13063* |
| 175 | 13063-R | GTTATTGTCCCCAGCCATAGGTC | R primer to amplify *MGG_13063* |
| 176 | 13283-F | GATGCGCATTACAACTTTGACAGC | F primer to amplify *MGG_13283* |
| 177 | 13283-NS-F | ATGACAGATGGGGGCCCAGTTATT | F primer to amplify *NS*-*MGG_13283* |
| 178 | 13283-R | CTTAATAACCCTTCCGTCTCCTGC | R primer to amplify *MGG_13283* |
| 179 | 13325-F | TATGAAGGCGCTTCTTTTAGTCTCC | F primer to amplify *MGG_13325* |
| 180 | 13325-R | CCTACTCTTCAGTGAATCCATCATTTGTG | R primer to amplify *MGG_13325* |
| 181 | 13863-F | TATGAAATGCAACAACATCATCCTC | F primer to amplify *MGG_13863* |
| 182 | 13863-R | CTTACATAATATTGCAGCCCTCTTCTC | R primer to amplify *MGG_13863* |
| 183 | 13872-F | TATGGCACCCTCACAACCACTC | F primer to amplify *MGG_13872* |
| 184 | 13872-R | TTCAGTACCGACTGTGGCTCCG | R primer to amplify *MGG_13872* |
| 185 | 14371-F | TATGGCATCGGCAGCAAGAT | F primer to amplify *MGG_14371* |
| 186 | 14371-NS-F | ATGCTCTACTGGCAGACAGC | F primer to amplify *NS*-*MGG_14371* |
| 187 | 14371-R | GTTTAGGCCCTGTTAGGCTCTG | R primer to amplify *MGG_14371* |
| 188 | 14652-F | TATGTTGCCCCAAACATTTCTCTT | F primer to amplify *MGG_14652* |
| 189 | 14652-R | GTCATCGCCCTTCTGACGG | R primer to amplify *MGG_14652* |
| 190 | 14725-F | AATGCAGATCTTCAAGATTGTTCAG | F primer to amplify *MGG_14725* |
| 191 | 14725-R | TTTAGCGATCATCACGAACTGTG | R primer to amplify *MGG_14725* |
| 192 | 14965-F | GATGAGGCCTTGCAGCTTTTTC | F primer to amplify *MGG_14965* |
| 193 | 14965-R | TTCACCATTCGTCATCAGAATCCT | R primer to amplify *MGG_14965* |
| 194 | 15022-F | AATGCGCTTCGCCACCATC | F primer to amplify *MGG_15022* |
| 195 | 15022-R | TCTCTAAGCGGAGCCGTCAATG | R primer to amplify *MGG_15022* |
| 195 | 15106-F | TATGCAGCTCGCCCGTTTTTATACT | F primer to amplify *MGG_15106* |
| 197 | 15106-R | ACTACCATTTTATCATTGTCGCATCTCC | R primer to amplify *MGG_15106* |
| 198 | 15371-F | TATGAGGCCTTGCAGCTTTTTCC | F primer to amplify *MGG_15371* |
| 199 | 15371-R | CTCACCATTCGTCATCAGAATCCTG | R primer to amplify *MGG_15371* |
| 200 | 15374-F | TATGCGCGCTTCGATTATTACCC | F primer to amplify *MGG_15374* |
| 201 | 15374-R | TCTAGGCGGCCTTGTTCTCATTCT | R primer to amplify *MGG_15374* |
| 202 | 15443-F | TATGCACGTTAAGCAATCGACTTT | F primer to amplify *MGG_15443* |
| 203 | 15443-R | TTTAACAGAGGGCCAACGTTCC | R primer to amplify *MGG_15374* |
|  | | | |
| 204 | MoActin-F | GTTCCTATTTACGAGGGTTTC | F primer for RT-PCR of *M. oryzae* *actin* |
| 205 | MoActin-R | GATGTCACGGACGATTTCT | R primer for RT-PCR of *M. oryzae* *actin* |
| 206 | CDIP6-RT-F | CTTTCCAGGGCCTGTTCG | F primer for RT-PCR of *MoCDIP6* |
| 207 | CDIP6-RT-R | GCGATGCCACCGATTTGC | R primer for RT-PCR of *MoCDIP6* |
| 208 | CDIP7-RT-F | CCTCGGTTGCCACCTTGA | F primer for RT-PCR of *MoCDIP7* |
| 209 | CDIP7-RT-R | ACTCGTCGTCGCAGTCCTC | R primer for RT-PCR of *MoCDIP7* |
| 210 | CDIP8-RT-F | TGCTCGCGTCCGAGACAA | F primer for RT-PCR of *MoCDIP8* |
| 211 | CDIP8-RT-R | ACCTTGATGCCCTCGCTCC | R primer for RT-PCR of *MoCDIP8* |
| 212 | CDIP9-RT-F | ATTACGCAACCCGCTCTG | F primer for RT-PCR of *MoCDIP9* |
| 213 | CDIP9-RT-R | TCAATTATCGGCAACCAAA | R primer for RT-PCR of *MoCDIP9* |
| 214 | CDIP10-RT-F | GCACAGTTCTGCGGTTTCAC | F primer for RT-PCR of *MoCDIP10* |
| 215 | CDIP10-RT-R | GCCAGGTTCTGAGGGATGAC | R primer for RT-PCR of *MoCDIP10* |
| 216 | CDIP11-RT-F | TGCCTGCTCGCCAAGTGC | F primer for RT-PCR of *MoCDIP11* |
| 217 | CDIP11-RT-R | GGCCGAGGCCGAAGAGTT | R primer for RT-PCR of *MoCDIP11* |
| 218 | CDIP12-RT-F | CAGCCGACAACGAAGTCA | F primer for RT-PCR of *MoCDIP12* |
| 219 | CDIP12-RT-R | GTATTTCGCCCTTGAGCC | R primer for RT-PCR of *MoCDIP12* |
| 220 | CDIP13-RT-F | GGCGGTTCACAGTCGCTCAA | F primer for RT-PCR of *MoCDIP13* |
| 221 | CDIP13-RT-R | GGCTCTGCAAAGTTCTTTCCCT | R primer for RT-PCR of *MoCDIP13* |
| 222 | OsActin-F | CTCAACCCCAAGGCTAACAG | F primer for RT-PCR of rice *Actin1* gene |
| 223 | OsActin-R | CCTTCATAGATTGGCACGGT | R primer for RT-PCR of rice *Actin1* gene |
| 224 | OsPR1b-F | ACGGGCGTACGTACTGGCTA | F primer for RT-PCR of rice *PR1b* gene |
| 225 | OsPR1b-R | CTCGGTATGGACCGTGAAG | R primer for RT-PCR of rice *PR1b* gene |
| 226 | OsNAC4-F | TCCTGCCACCATTCTGAGATG | F primer for RT-PCR of rice *Nac4* gene |
| 227 | OsNAC4-R | TTGCAGAATCATGCTTGCCAG | R primer for RT-PCR of rice *Nac4* gene |
| 228 | OsCHT1-F | GCACTGATAACCACTGATCGG | F primer for RT-PCR of rice *CHT1* gene |
| 229 | OsCHT1-R | TGTGGGCATTACTGATGATTG | R primer for RT-PCR of rice *CHT1* gene |
| 230 | OsCHT3-F | GCGATAACCTGGATTGCTACAACC | F primer for RT-PCR of rice *CHT3* gene |
| 231 | OsCHT3-R | GTATTTTATTCGTCTGCTCGG | R primer for RT-PCR of rice *CHT3* gene |
|  | | | |
| 232 | YS-6-F | CCGGAATTCATGAAGTTCTCCGCCATC | F primer of *FL-CDIP6* for yeast secretion |
| 233 | YS-6-NS-F | CCGGAATTCATGGCTCCCACCAGC | F primer of *NS-CDIP6* for yeast secretion |
| 234 | YS-6-R | ATTGCGGCCGCAACAGGATGAAACC | R primer for *CDIP6* for yeast secretion |
| 235 | YS-7-F | CCGGAATTCATGCGCTCTGCCATGATC | F primer of *FL-CDIP7* for yeast secretion |
| 236 | YS-7-NS-F | CCGGAATTCATGCACCCTCCCAAGC | F primer of *NS-CDIP7* for yeast secretion |
| 237 | YS-7-R | ATTGCGGCCGCAACAAGAAAGCAACG | R primer for *CDIP7* for yeast secretion |
| 238 | YS-8-F | CCGGAATTCATGGCCTCCCTCCCCCT | F primer of *FL-CDIP8* for yeast secretion |
| 239 | YS-8-R | ATTGCGGCCGCAATCGAAGAATCACGT | R primer for *CDIP8* for yeast secretion |
| 240 | YS-9-F | CCGGAATTCATGCGCATCACCAGCCG | F primer of *FL-CDIP9* for yeast secretion |
| 241 | YS-9-NS-F | CCGGAATTCATGCCGACCGGCTTGCT | F primer of *NS-CDIP9* for yeast secretion |
| 242 | YS-9-R | ATTGCGGCCGCAAGTCCTTCTTCTGGGTTT | R primer for *CDIP9* for yeast secretion |
| 243 | YS-10-F | CCGGAATTCATGCCTTCCATCATCAAGA | F primer of *FL-CDIP10* for yeast secretion |
| 244 | YS-10-NS-F | CCGGAATTCATGCTCCCCTCACAGTCGA | F primer of *NS-CDIP10* for yeast secretion |
| 245 | YS-10-R | ATTGCGGCCGCAAGAGAGAGCCAGGA | R primer for *CDIP10* for yeast secretion |
| 246 | YS-11-F | CCGGAATTCATGAAGTTCTCCATCATC | F primer of *FL-CDIP11* for yeast secretion |
| 247 | YS-11-NS-F | CCGGAATTCATGCAGGACCTCTCTTCC | F primer of *NS-CDIP11* for yeast secretion |
| 248 | YS-11-R | ATTGCGGCCGCAACAAAGCAAGGAC | R primer for *CDIP11* for yeast secretion |
| 249 | YS-12-F | CCGGAATTCATGCGCATTACAACTTTGA | F primer of *FL-CDIP12* for yeast secretion |
| 250 | YS-12-NS-F | CCGGAATTCATGACAGATGGGGGCCCA | F primer of *NS-CDIP12* for yeast secretion |
| 251 | YS-12-R | ATTGCGGCCGCAAATAACCCTTCCGTCT | R primer for *CDIP12* for yeast secretion |
| 252 | YS-13-F | TATGGCATCGGCAGCAAGAT | F primer of *FL-CDIP13* for yeast secretion |
| 253 | YS-13-NS-F | CCGGAATTCATGCTCTACTGGCAGACA | F primer of *NS-CDIP13*for yeast secretion |
| 254 | YS-13-R | ATGCTCTACTGGCAGACAGC | R primer for *CDIP13* for yeast secretion |
|  | | | |
| 255 | CDIP6-UA-F | CCCACCCAGCCATACCCA | F primer for identifying Δ-MoCDIP6 mutant |
| 256 | CDIP6-AF-*Sa*lI | AGTCGACATGCCACGGGTCTCCACGAT | Primer for making Δ-MoCDIP6 mutant |
| 257 | CDIP6-AR-*Eco*RI | TGAATTCGGAGTGAAGCGAACAACG | Primer for making Δ-MoCDIP6 mutant |
| 258 | CDIP6-BF-*Spe*I | GACTAGTAACCATCTCCGCTTCTAT | Primer for making Δ-MoCDIP6 mutant |
| 259 | CDIP6-BR-*Xba*I | GTCTAGATCATCCTTTGTTACTACGC | Primer for making Δ-MoCDIP6 mutant |
| 260 | CDIP7-UA-F | GCGAAACTGACCAATACG | F primer for identifying Δ-MoCDIP7 mutant |
| 261 | CDIP7-AF-*Sal*I | AGTCGACTCAGGGTCACCATACCGA | Primer for making Δ-MoCDIP7 mutant |
| 262 | CDIP7-AR-*Eco*RI | GGAATTCAAATCAGGCTGGCATCTA | Primer for making Δ-MoCDIP7 mutant |
| 263 | CDIP7-BF-*Spe*I | CACTAGTTTGTTGGGTGTTGGTTCT | Primer for making Δ-MoCDIP7 mutant |
| 264 | CDIP7-BR-*Xba*I | ATCTAGAAGGCTGGCTGGAATGAGA | Primer for making Δ-MoCDIP7 mutant |
| 265 | CDIP8-UA-F | CATCACGAGTCTTCCCAACA | F primer for identifying Δ-MoCDIP8 mutant |
| 266 | CDIP8-AF-*Kpn*I | TGGTACCGGCGATCTGATGTTTGGA | Primer for making Δ-MoCDIP8 mutant |
| 267 | CDIP8-AR-*Sal*I | AGTCGACAAGGAGTGTAACTGCGTGT | Primer for making Δ-MoCDIP8 mutant |
| 268 | CDIP8-BF-*Spe*I | GACTAGTTGCTGTAAATTAAGCCTTGC | Primer for making Δ-MoCDIP8 mutant |
| 269 | CDIP8-BR-*Xba*I | ATCTAGAGGTCGTCTGCCGTCACTA | Primer for making Δ-MoCDIP8 mutant |
| 270 | CDIP9-UA-F | CACAAACAAGCCATAAAC | F primer for identifying Δ-MoCDIP9 mutant |
| 271 | CDIP9-AF-*Xho*I | TGGTACCGAACATGCGTGGCTTTGC | Primer for making Δ-MoCDIP9 mutant |
| 272 | CDIP9-AR-*Eco*RI | GAAGCTTCGAGGAAGTTAGGGACAT | Primer for making Δ-MoCDIP9 mutant |
| 273 | CDIP9-BF-*Spe*I | AACTAGTCGCCAGGCTCACCATACT | Primer for making Δ-MoCDIP9 mutant |
| 274 | CDIP9-BR-*Sac*I | ATCTAGACATTCACCAGGTCCGTTC | Primer for making Δ-MoCDIP9 mutant |
| 275 | CDIP10-UA-F | CTCAGTGGCGGGCTATTA | F primer for identifying Δ-MoCDIP10 mutant |
| 276 | CDIP10-AF-*Kpn*I | AGGTACCGTAGAGCGTACCACAGGC | Primer for making Δ-MoCDIP10 mutant |
| 277 | CDIP10-AR-*Hind*III | AGTCGACCGTGAGCGAGGGTGATTT | Primer for making Δ-MoCDIP10 mutant |
| 278 | CDIP10-BF-*Spe*I | CACTAGTATATTGGGCATTGTGGTG | Primer for making Δ-MoCDIP10 mutant |
| 279 | CDIP10-BR-*Xba*I | CTCTAGAAAGGACGATAGAGGAGCA | Primer for making Δ-MoCDIP10 mutant |
| 280 | CDIP11-UA-F | CGATTCGTGATTGGGTTTA | F primer for identifying Δ-MoCDIP11 mutant |
| 281 | CDIP11-AF-*Kpn*I | AGTCGACGCTTATGAGTCTGGAGGTTG | Primer for making Δ-MoCDIP11 mutant |
| 282 | CDIP11-AR-*Sal*I | TGAATTCTAGGCAAGGAGAATGACG | Primer for making Δ-MoCDIP11 mutant |
| 283 | CDIP11-BF-*Spe*I | GACTAGTGAGATTGGATGGCGTTTC | Primer for making Δ-MoCDIP11 mutant |
| 284 | CDIP11-BR-*Xba* I | TGAGCTCCAGTGTAGGTCCGGGATA | Primer for making Δ-MoCDIP11 mutant |
| 285 | CDIP12-UA-F | AGGTTCGGGTCCGTTGGT | F primer for identifying Δ-MoCDIP12 mutant |
| 286 | CDIP12-AF-*Sal*I | AGTCGACCCTCAGAGCCATTCTTTCG | Primer for making Δ-MoCDIP12 mutant |
| 287 | CDIP12-AR-*Eco*RI | TGAATTCAGTTCACTGACGCGACCC | Primer for making Δ-MoCDIP12 mutant |
| 288 | CDIP12-BF-*Spe*I | AGGATCCATTCCCGAGTCTCCACCAA | Primer for making Δ-MoCDIP12 mutant |
| 289 | CDIP12-BR-*Sac*I | TGAGCTCATTTACAAGCACCTCACATA | Primer for making Δ-MoCDIP12 mutant |
| 290 | CDIP13-UA-F | GCGGGTGACCTGTTTGAC | F primer for identifying Δ-MoCDIP13 mutant |
| 291 | CDIP13-AF-*Sal*I | CGGTACCCGACGGTCTTTACGGATTG | Primer for making Δ-MoCDIP13 mutant |
| 292 | CDIP13-AR-*Eco*RI | CGAATTCGGTACTTGGCTAACAGTGAATT | Primer for making Δ-MoCDIP13 mutant |
| 293 | CDIP13-BF-*Bam*HI | GACTAGTTCCGATGTGGGAACCTTA | Primer for making Δ-MoCDIP13 mutant |
| 294 | CDIP13-BR-*Sac*I | GTCTAGACAGTTAGAAACCCTTGACCT | Primer for making Δ-MoCDIP13 mutant |
